# Ascorbic acid induces salivary gland function through TET2/acetylcholine receptor signaling in aging SAMP1/Klotho (-/-) mice

**DOI:** 10.18632/aging.204213

**Published:** 2022-08-11

**Authors:** Nguyen Khanh Toan, Soo-A Kim, Sang-Gun Ahn

**Affiliations:** 1 Department of Pathology, School of Dentistry, Chosun University, Gwangju 61452, Republic of Korea; 2 Department of Biochemistry, School of Oriental Medicine, Dongguk University, Gyeongju 38066, Republic of Korea

**Keywords:** aging, salivary gland, ascorbic acid, AchRs, DNA demethylase

## Abstract

Aging affects salivary gland function and alters saliva production and excretion. This study aimed to investigate whether ascorbic acid can be used to treat salivary gland dysfunction in an extensive aging mouse model of SAMP1/Klotho-/- mice. In our previous study, we found that ascorbic acid biosynthesis was disrupted in the salivary glands of SAMP1/Klotho (-/-) mice subjected to metabolomic profiling analysis. In SAMP1/Klotho -/- mice, daily supplementation with ascorbic acid (100 mg/kg for 18 days) significantly increased saliva secretion compared with the control. The expression of salivary gland functional markers (α-amylase, ZO-1, and Aqua5) is upregulated. Additionally, acetylcholine and/or beta-adrenergic receptors (M1AchR, M3AchR, and Adrb1) were increased by ascorbic acid in the salivary glands of aging mice, and treatment with ascorbic acid upregulated the expression of acetylcholine receptors through the DNA demethylation protein TET2. These results suggest that ascorbic acid could overcome the lack caused by dysfunction of ascorbic acid biosynthesis and induce the recovery of salivary gland function.

## INTRODUCTION

Aging is an inevitable event in every living organism, and it is associated with a loss of metabolic homeostasis with multiple pathophysiological processes [1]. In humans, salivary glands exhibit an age-dependent and medication-independent loss of function in the elderly population, resulting in a decrease in resting saliva volume [2]. It is often suggested that the salivary gland dysfunction with age results in dry mouth conditions, defective teeth, and poor oral hygiene. Based on recent studies, saliva viscosity, lubrication, and saliva components, including several enzymes, hormones, antioxidants, growth factors, and antimicrobial substances, are decreased in the saliva of aged populations [3–5]. The impact of aging on salivary glands is demonstrated in both histological and functional aspects. The epithelial secretory structures of salivary glands are reduced and replaced by adipose and fibrous tissue and show salivary gland hyposalivation in several aging mouse models [6, 7]. These deteriorations imply a great reduction in oral health, as saliva plays a core role in maintaining tooth and oral protection. Therefore, a new approach that can reverse aging-induced salivary gland dysfunction needs to be developed.

Ascorbic acid (AA) is a powerful antioxidant that mediates several beneficial effects on oxidative stress, the immune system, inflammation, aging, and metabolism [8–12]. Ascorbic acid is also a major cofactor for numerous enzymes involved in the biosynthesis of collagen and carnitine, conversion of dopamine to noradrenaline, and tyrosine metabolism and participates in epigenetic regulation [13–16]. An imbalance in ascorbic acid homeostasis has been demonstrated in aging diseases, including neurodegenerative diseases [17]. Therefore, during the aging-related disease process, a clear link exists between ascorbic acid metabolism/homeostasis and aging disorders. *In vivo* studies have suggested that ascorbic acid prevents aging-related diseases and chronic diseases and rescues lifespan in several aging models, including Werner’s syndrome, SAMP6 (senescence accelerated mice prone 6), SMP30KO (senescence marker protein-30 knockout), and Gulo-deficient mouse models [11, 17–20]. In the elderly population, high-dose ascorbic acid reduces oxidative stress by lipid peroxidation and proinflammatory cytokines and amyloid plaque formation in Alzheimer’s disease patients [21]. A previous study found that ascorbic acid deficiency is involved in the loss of salivary secretion, with swelling of the major salivary glands [22]. In addition, in an *in vivo* model, ascorbic acid-deficient animals reduced stimulated whole salivary flow rates by impaired muscarinic acetylcholine receptors and β-adrenergic receptor signaling [23].

In our previous study, we analyzed the metabolic profile in salivary glands from aging-accelerated mice [24]. In metabolic pathway analysis, various metabolic abnormalities are induced in the aging mice, such as hypoglycemia, lack of glycogen storage, low fat in brown adipose tissue as well as acetylcholine (Ach) metabolites [24, 25]. Interestingly, we found that the levels of ascorbic acid were relatively lower in the salivary glands of aging mice than in those of wild-type mice. We also demonstrated that the levels of metabolites associated with glutathione (GSH) metabolism differed in the salivary glands of aging-accelerating mice [26]. Therefore, we hypothesize that there might be a connection between aging-induced salivary gland dysfunction and ascorbic acid metabolism disruption.

In this study, we investigated ascorbic acid metabolic alterations that may be related to salivary gland dysfunction in aging SAMP1/kl-/- mice. We found that aging SAMP1/kl-/- mice exhibit lower saliva volume and impaired ascorbic acid metabolism than wild-type mice. This alteration is linked to a decrease in the expression of Ten-eleven translocation methyl-cytosine dioxygenase 2 (TET2). Ascorbic acid improved salivary gland function and induced TET2 expression in SAMP1/kl-/- mice. Consistently, in PSGC +/+ cells, TET2 overexpression restored the expression of muscarinic (M1/M3 AchR) and adrenergic receptors (β-adrenergic receptors) and improved the functional marker proteins of the salivary gland. The age-related alteration of ascorbic acid metabolism in aging mice might also account for the concomitant changes between epigenetic (DNA methylation) approaches and salivary gland function. These results suggest that the ascorbic acid pathway might have therapeutic benefits for the treatment of age-related salivary gland dysfunction.

## RESULTS

### Ascorbic acid biosynthesis is decreased in salivary glands of aging SAMP/Kl -/- mice

**Figure 1:**
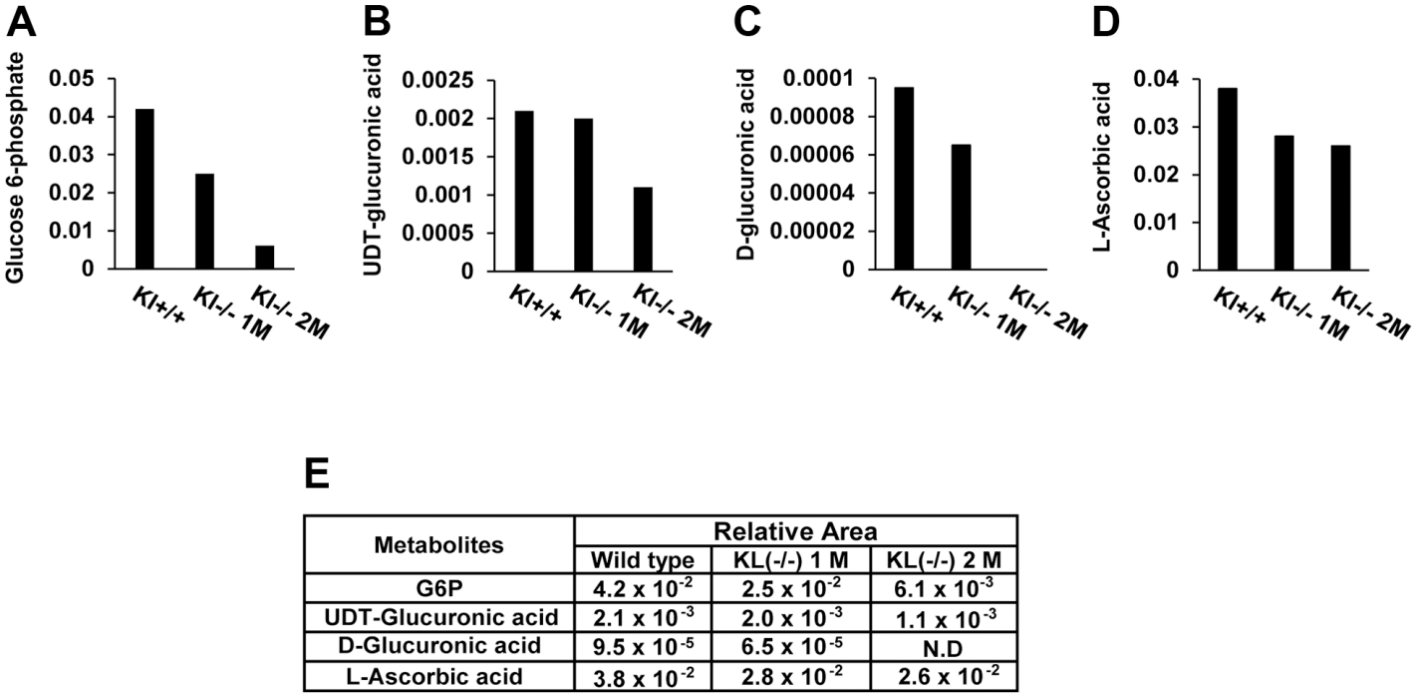
**A comparison of the metabolites of ascorbic acid biosynthesis in aging mouse salivary glands.** Metabolic profiling was conducted by CE-TOFMS using aging-accelerated mice (SAMP1/kl+/+, SAMP1/kl-/- 1 month, and SAMP1/kl-/- 2 months) (**A**–**D**) Changes in intermediates in ascorbic acid metabolism. The relative quantities of detected metabolites are represented as bar graphs (glucose 6-phosphate, UDP-glucuronic acid, D-glucuronic acid, and L-ascorbic acid). (**E**) Comparisons of the relative amount of ascorbic acid metabolites between SAMP1/kl+/+, SAMP1/kl-/- 1-month-old, and SAMP1/kl-/- 2-month-old mouse salivary glands.

Previously, we conducted a metabolome analysis in salivary glands of aging SAMP1/kl-/- mice with wild-type SAMP1/kl-/- mice and illustrated the metabolites related to aging-mediated salivary gland dysfunction [25]. In this study, we found a noticeable difference between several metabolites in the ascorbic acid biosynthesis pathway, including UDP-glucuronic acid, D-glucuronic acid, and L-ascorbic acid (Figure 1A–1E), in aging SAMP1/kl -/- mice. As shown in Figure 1E, ascorbic acid (vitamin C, ascorbate) levels were reduced by approximately 32% in salivary glands of 2-month-old SAMP1/kl-/- mice compared with wild-type mice; similarly, their precursors were also significantly reduced. The UDP-glucuronic acid and glucose 6-phosphate levels were decreased by 48% and 85%, respectively, while the D-glucuronic acid level diminished completely when SAMP1/kl-/- mice reached 2 months of age. Therefore, these data suggested that the ascorbic acid biosynthesis pathway in the aging SAMP1 kl -/- model may be impaired.

### Ascorbic acid rescues muscarinic and adrenergic receptors in salivary glands

**Figure 2:**
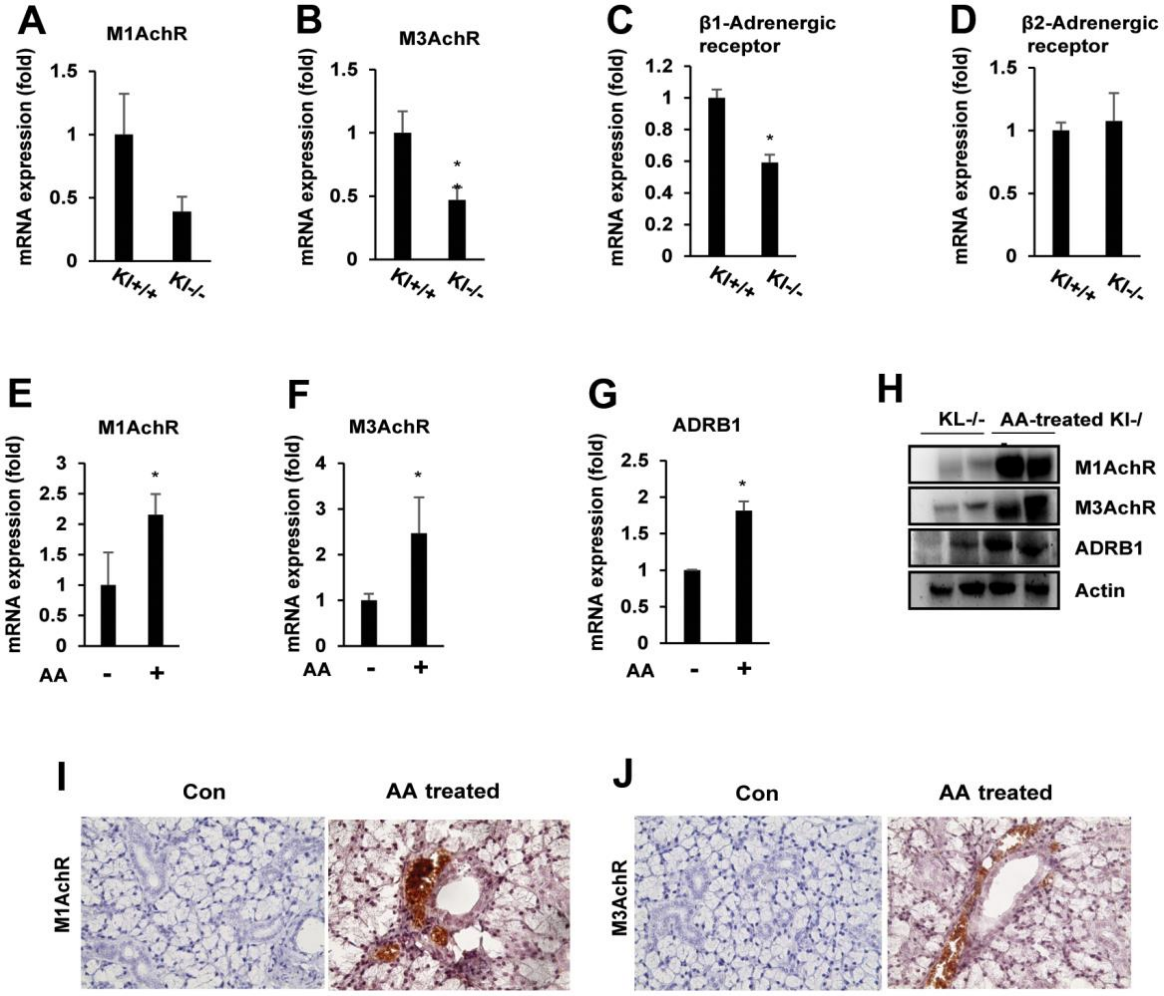
**Ascorbic acid upregulates muscarinic and adrenergic receptors in aging SAMP1/kl -/- mice.** (**A**–**D**) Age-related muscarinic (M1/M3AchR) and adrenergic receptor (ADRβ1/β2) alterations in the salivary gland. Total mRNA was isolated from salivary gland tissues of SAMP1/kl+/+ and SAMP1/kl-/- mice. Quantification of endogenous mRNA expression was performed by quantitative reverse transcription-polymerase chain reaction (qRT–PCR). *p < 0.05. (**E**–**H**) Effects of ascorbic acid treatment in the salivary glands of SAMP1/kl -/- mice. SAMP1/kl -/- mice were fed 100 mg/kg ascorbic acid orally daily. Muscarinic and adrenergic receptor expression in the salivary glands of SAMP1/kl-/- mice treated with ascorbic acid was analyzed by quantitative RT–PCR or Western blotting. *p < 0.05. (**I**, **J**) Immunohistochemistry staining of M1 and M3AchR in tissue sections of salivary glands from ascorbic acid-treated SAMP1/kl -/- mice. Data are presented as the mean ± standard deviation (SD). Each group was compared with the mean of the control.

Several studies have suggested that ascorbic acid deficiency reduces muscarinic and adrenergic receptors in salivary glands as well as the downstream cellular signaling pathways [23, 27]. Therefore, we evaluated the expression of muscarinic and adrenergic receptors in salivary glands of aging SAMP1/kl-/- mice compared with wild-type mice using qRT–PCR. We found that the expression of muscarinic receptors M1 (M1AchR) and M3 (M3AchR) was significantly inhibited in aging SAMP1/kl-/- mice (Figure 2A, 2B). In the adrenergic receptor family, there was a noticeable reduction in the mRNA expression of adrenergic receptor β1, while no significant difference was detected in the case of adrenergic receptor β2 (Figure 2C, 2D).

To investigate the role of ascorbic acid on the expression of muscarinic and adrenergic receptors in salivary glands, we treated aging SAMP1/kl-/- mice with ascorbic acid at a dose of 100 mg/kg orally and then examined the expression of muscarinic and adrenergic receptors in salivary glands. As shown in Figure 2E–2H, we found that ascorbic acid significantly induced the mRNA and protein expression of muscarinic receptor M1, M3, and adrenergic receptor β1. In the immunohistochemical analysis, we also observed muscarinic receptor M1 and M3 immunoreactivity in the salivary gland (Figure 2I, 2J). These results suggested the positive roles of ascorbic acid on the expression of salivary gland receptors.

### Ascorbic acid induces salivary gland function in SAMP1/kl-/- mice

**Figure 3:**
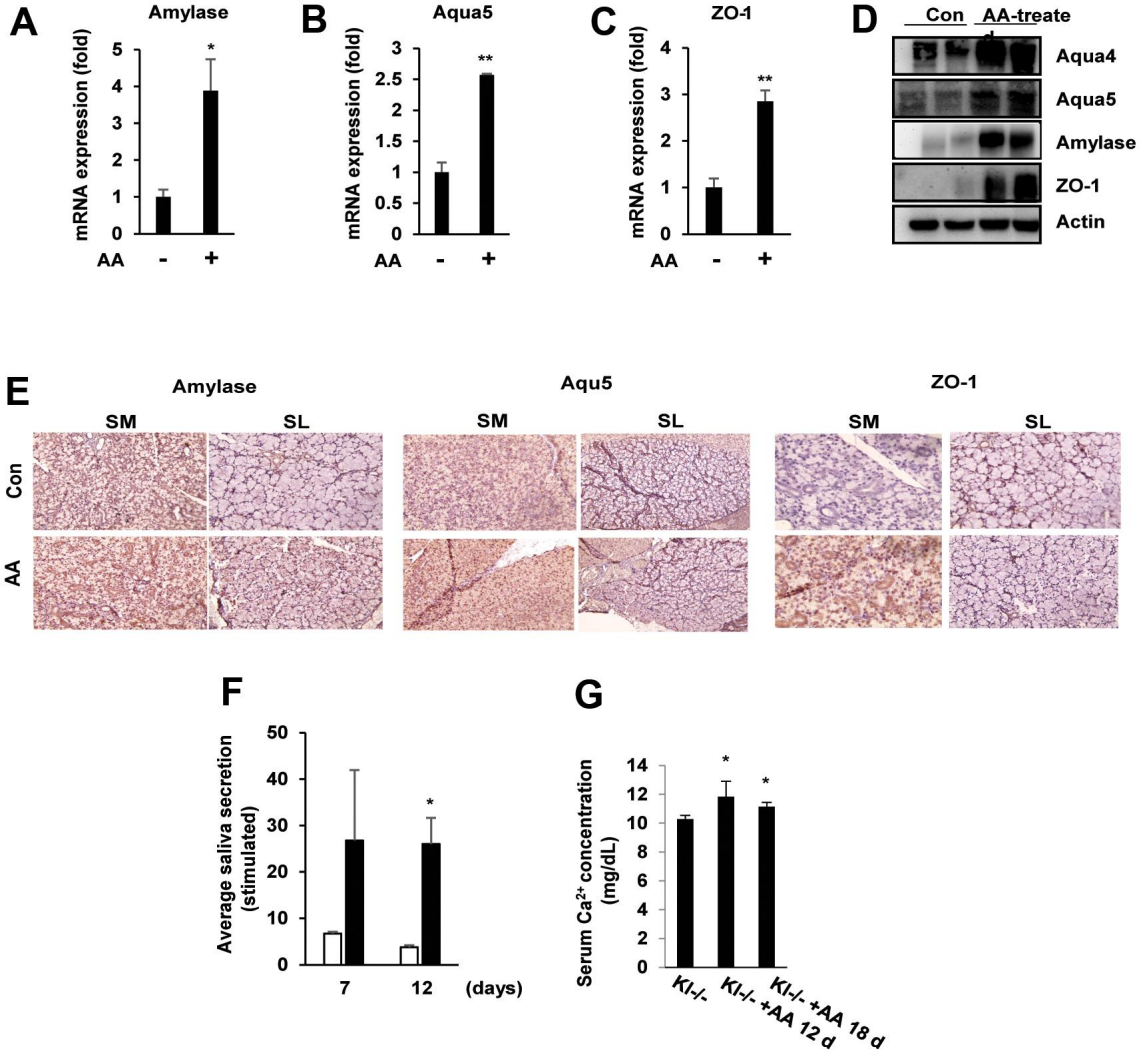
**Ascorbic acid induces the expression of salivary gland functional proteins involved in saliva secretion.** SAMP1/kl -/- mice were fed daily with ascorbic acid (100 mg/kg). (**A**–**D**) α-amylase, aqua4/5, and ZO-1 expression in the salivary glands of SAMP1/ kl-/- mice was analyzed by quantitative RT–PCR or Western blotting. *p < 0.05, **p < 0.01. (**E**) Immunohistochemistry staining of α-amylase, aqua4/5, and ZO-1 in submandibular or sublingual tissue of salivary glands from ascorbic acid-treated SAMP1/ kl -/- mice. (**F**) After oral administration of ascorbic acid in SAMP1/kl-/- mice, saliva was stimulated with an acetylcholine injection at 7 or 12 days. (**G**) Serum Ca^2+^ levels in the control and ascorbic acid-treated SAMP1/kl-/- mice at the indicated times. *p < 0.05. Data are presented as the mean ± standard deviation (SD). Each group was compared with the mean of the control.

To determine whether ascorbic acid expresses functional markers of salivary glands, quantitative reverse transcription PCR (qRT–PCR) was performed to validate the expression of these genes in the salivary glands of ascorbic acid-treated aging mice. α-amylase (AC secretion product), aquaporin 5 (water channel protein), and ZO-1 (tight junction protein), which are specifically expressed in the mammalian salivary gland, were significantly induced in ascorbic acid-treated aging mice compared with aging SAMP1/kl -/- mice (Figure 3A–3C). Consistent with the mRNA expression results, the protein expression of α-amylase, aquaporin-4, aquaporin-5, and ZO-1 was also confirmed by immunoblotting (Figure 3D). These results were confirmed by immunohistochemistry staining, which showed the upregulation of all three proteins in both submandibular and sublingual salivary glands (Figure 3E). In addition, we found that the stimulated saliva secretion in ascorbic acid-treated aging SAMP1/kl-/- mice increased by approximately 5 times after 12 days of treatment compared with nontreated SAMP1/kl-/- mice (Figure 3F). Finally, the level of ion Ca^2+^ in the serum of ascorbic acid-treated aging SAMP1/kl-/- mice was also increased significantly (Figure 3G). These results suggested that ascorbic acid improves salivary gland function in SAMP1/kl-/- mice.

### Ascorbic acid induces α-amylase, aquaporin-4, aquaporin-5, and ZO-1 expression in mouse primary salivary gland cell (PSGC) lines

**Figure 4:**
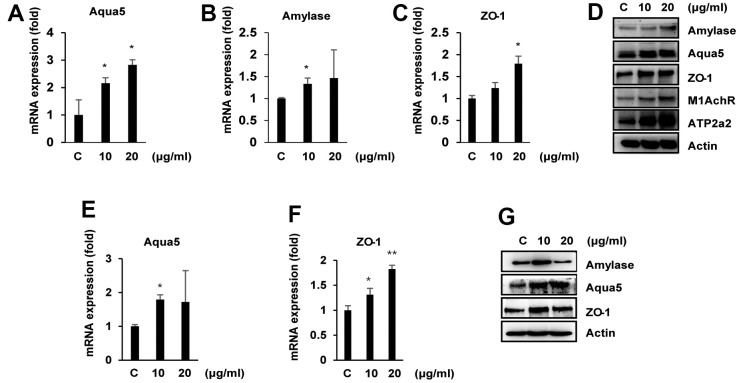
**Effects of ascorbic acid on primary salivary gland cells (PSGCs) isolated from SAMP1/kl +/+ and SAMP1/kl-/- mice.** (**A**–**D**) PSGS kl+/+ cells isolated from SAMP1/kl +/+ mice were treated with ascorbic acid at the indicated concentrations for 24 h. mRNA and protein expression of salivary gland functional proteins in PSGC kl+/+ cells were determined by qRT–PCR and Western blotting. *p < 0.05. (**E**–**G**). Under the same conditions, the mRNA and protein expression of several salivary gland functional proteins under ascorbic acid treatment in PSGC kl-/- cells isolated from SAMP1/kl-/- mice. mRNA and protein expression were determined by qRT–PCR and Western blotting, respectively. *p < 0.05; **p < 0.01 versus the control.

We first assessed the effects of ascorbic acid on α-amylase, aquaporin-4, aquaporin-5, and ZO-1 expression in the mouse primary salivary gland cell lines PSGC kl +/+ and PSGC kl -/-. As shown in Figure 4A–4C, ascorbic acid upregulated the mRNA levels of α-amylase, aquaporin-4, aquaporin-5, and ZO-1 in PSGC kl +/+ cells in a concentration-dependent manner. Additionally, total protein was extracted after ascorbic acid treatment and used to determine α-amylase, aquaporin-4, aquaporin-5, and ZO-1 levels by Western blotting. Consistently, ascorbic acid induced α-amylase, aquaporin-4, aquaporin-5, and ZO-1 protein expression in a similar pattern as mRNA (Figure 4D), indicating that induction of α-amylase, aquaporin-4, aquaporin-5, and ZO-1 expression by ascorbic acid can be at the transcriptional level. ATP2A2 was used as a positive control for ascorbic acid. Similar to PSGC kl +/+ cells, ascorbic acid also induced α-amylase, aquaporin-4, aquaporin-5, and ZO-1 protein and mRNA expression in PSGC kl -/- cells (Figure 4E–4G). In addition, we observed that cell viability and proliferation were not altered by ascorbic acid in the range of 20-100 μg/ml (Supplementary Figure 1).

### Ascorbic acid rescues TET2 expression in SAMP1/kl-/- mice

**Figure 5:**
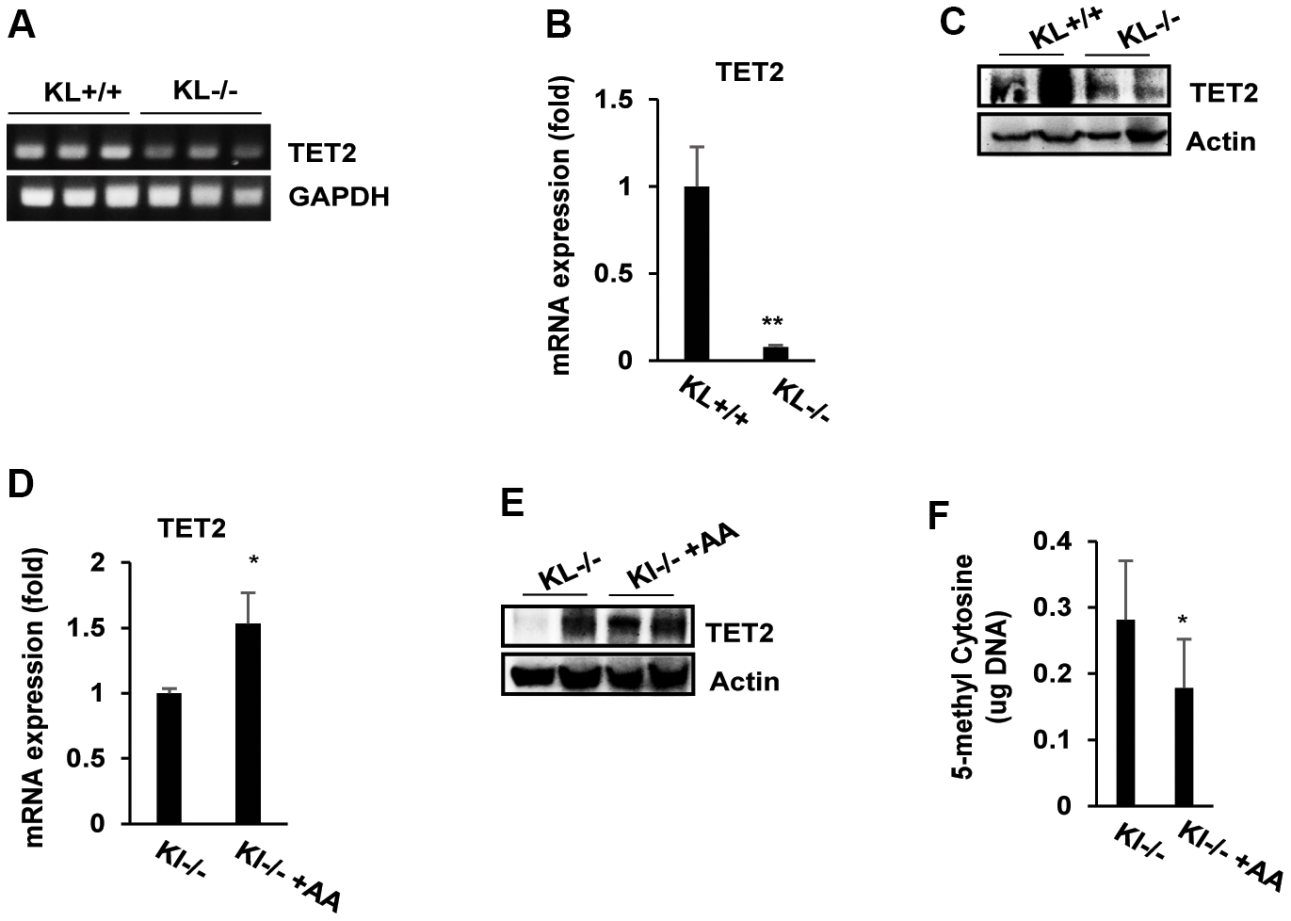
**The expression of TET2 and DNA methylation status in ascorbic acid-treated SAMP1/kl-/- mice.** (**A**, **B**) Endogenous TET2 mRNA expression in the salivary glands of SAMP1/kl+/+ and SAMP1/kl-/- mice was measured by RT–PCR or qRT–PCR. GAPDH was used as an internal control. **p < 0.01. (**C**) The relative expression levels of TET2 protein in the salivary glands between SAMP1/kl +/+ and SAMP1/kl -/- mice were analyzed by Western blotting. (**D**, **E**) SAMP1/kl -/- mice (4 weeks old) were randomly divided into two groups (5 mice/group) and orally injected with ascorbic acid (100 mg/kg) or the same volume of saline for 18 days. After treatment, mouse salivary glands were collected and used for the following assays. The expression of TET2 mRNA and protein was determined by qRT–PCR (**D**) and Western blotting (**E**). (**F**) Quantitative analysis of 5-mC levels. Total 5-mC levels in salivary glands isolated from SAMP1/kl -/- mice and SAMP1/kl -/- mice treated with ascorbic acid were measured. *p < 0.05.

To identify the molecular mechanism by which ascorbic acid induces muscarinic receptor M1 and M3 expression in salivary glands, we investigated the effects of ascorbic acid on regulatory factors responsible for muscarinic receptor M1 and M3 expression/activation, such as the ten-eleven-translocation (TET) family. Therefore, we identified the expression of the TET family gene in salivary glands of SAMP1/kl +/+ and SAMP1/kl -/- mice. Among the TET family members, only TET2 was detected in salivary glands, while TET1 and TET3 were not detected (data not shown). Compared with SAMP1/kl +/+ mice, SAMP1/kl -/- mice showed reduced TET2 expression of more than 80% in the salivary gland (Figure 5A, 5B). TET2 protein levels were also decreased in SAMP1/kl -/- mice (Figure 5C), which resulted in TET2 reduction in the salivary glands of SAMP1/kl -/- mice.

To determine the effects of ascorbic acid on TET2 expression, SAMP1/kl-/- mice were orally administered ascorbic acid solution or vehicle (saline) daily for 18 days. As shown in Figure 5D, ascorbic acid supplementation moderately increased TET2 mRNA levels in the salivary gland. Compared with SAMP1/kl -/- mice, ascorbic acid treatment also increased the levels of TET2 protein in SAMP1/kl -/- mice (Figure 5E).

Ascorbic acid has also been reported to induce global demethylation *in vivo* through the expression/activation of the TET family [28]. DNA demethylation plays important role in the processes of development and differentiation. TET2 is a protein that catalyzes the conversion of the modified DNA base 5-methylcytosine (5-mC) to 5-hydroxymethylcytosine (5-hmC). Therefore, we determined the effect of ascorbic acid on DNA demethylation. Treatment of SAMP1/kl-/- mice with ascorbic acid significantly decreased 5-methylcytosine (Figure 5F). Taken together, the results above suggest that ascorbic acid induces global demethylation by enhancing TET2 expression/activity in salivary glands.

### Overexpression of TET2 upregulates salivary gland functional markers

**Figure 6:**
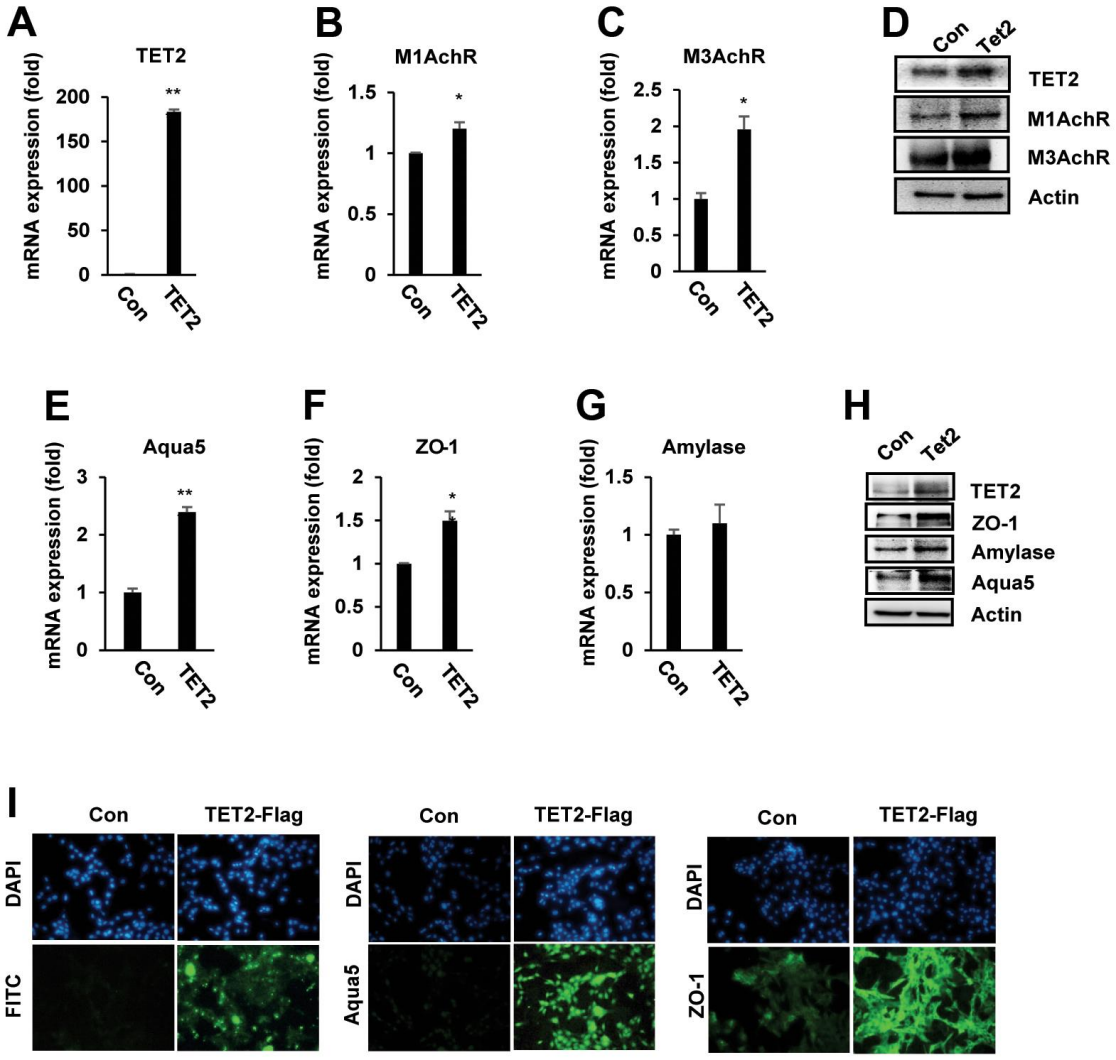
**TET2 upregulated muscarinic receptor and salivary gland functional protein expression in PSGC kl +/+ cells.** PSGC kl +/+ cells were transfected with the TET2 expression plasmid (pcDNA3-TET2-Flag) for 48 hrs. After transfection, qRT–PCR and Western blotting were performed to evaluate the mRNA and protein levels. (**A**–**D**) Expression of M1 and M3 muscarinic receptors (M1 and M3AchR) in PSGC kl +/+ cells overexpressing TET2. (**E**–**H**) Effect of TET2 on α-amylase, aqua5, and ZO-1 expression. Data are presented as the mean ± SD. *p < 0.05, **p < 0.01. (**I**) Immunocytochemistry staining of ZO-1 and Aqua5 in TET2-overexpressing PSGC kl +/+ cells. PSGC kl +/+ cells were transfected as above for 48 h. After transfection, anti-mouse Flag antibody and Alexa Flour 488-labeled sheep anti-mouse secondary antibody were used to detect TET2 proteins. Aqua5 and ZO-1 were detected by primary anti-rabbit Aqua5 and ZO-1 antibodies and Alexa Flour 488-labeled sheep anti-rabbit secondary antibodies (**I**). Bar: 100 μm. Images were observed with a confocal microscope.

To verify the role of TET2 on muscarinic receptors (M1AchR and M3AchR) and salivary gland functional marker expression, we overexpressed TET2 in PSGC kl +/+ cells and observed their gene expression upon TET2 overexpression. *TET2* mRNA expression was upregulated in *TET2*-overexpressing PSGC kl +/+ cells compared with control cells (Figure 6A). qRT–PCR and Western blotting analyses showed that TET2 overexpression significantly upregulated M1AchR and M3AchR expression in PSGC kl +/+ cells (Figure 6B–6D). Importantly, overexpression of TET2 also induced the mRNA expression of amylase, Aqua5, and ZO-1, indicating regulation of salivary gland function (Figure 6D–6G). Western blot analysis confirmed the increase in amylase, Aqua5, and ZO-1 in *TET2*-overexpressing PSGC kl +/+ cells (Figure 6H). In immunocytochemistry analysis, we also confirmed that the levels of the tight junction protein ZO-1 and water channel Aqua5 in TET2-overexpressing cells were correspondingly higher than those in vesicle-transfected control cells (Figure 6I).

### Knockdown of TET2 decreases the expression of salivary gland function associated genes

**Figure 7:**
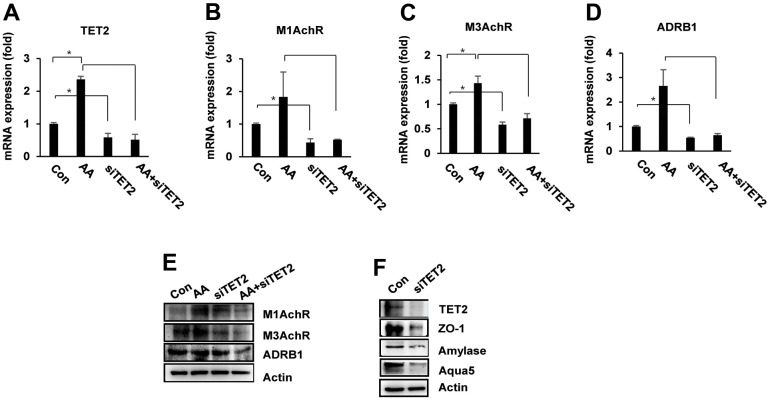
**TET2 knockdown impairs ascorbic acid-mediated salivary gland functional activity.** PSGC kl +/+ cells were transfected with siTET2 for 24 hrs. Cells were then treated with ascorbic acid (20 μg/mL) and incubated for 24 hrs, and mRNA expression was evaluated by qRT–PCR. (**A**–**D**) mRNA expression of M1 AchR, M3AchR, and ADRB1 in PSGC kl +/+ cells treated with ascorbic acid, siRNA TET2, or both. Data are presented as the mean ± SD. *p < 0.05, **p < 0.01. (**E**) The expression of M1AchR, M3AchR, and ADRB1 protein was determined by Western blotting under the same conditions. (**F**). Effects of siTET2 on salivary gland functional proteins. PSGC kl +/+ cells were transfected with siTET2 for 48 hrs. After transfection, the protein levels of α-amylase, aqua5, and ZO-1 were analyzed by Western blotting. Actin was used as an internal control.

To investigate whether TET2 mediates the muscarinic receptor-mediated pathway and DNA demethylation of its promoter, we knocked down TET2 expression in PSGC kl +/+ cells using small interfering RNAs (siRNAs). First, we evaluated the effect of TET2 inhibition with or without ascorbic acid. When transfected into cells, TET2 siRNAs specifically decreased the mRNA levels of *TET2* to below 50% compared with the control (Figure 7A). qRT–PCR analysis showed that muscarinic and adrenergic receptors, including M1AchR, M3AchR, and ADRβ1, were significantly elevated in ascorbic acid-treated PSGC kl +/+ cells compared with the control cells without ascorbic acid-PSGC kl +/+. We found that TET2 siRNA led to significantly decreased M1AchR, M3AchR, and ADRβ1 expression when compared with the control. Furthermore, we evaluated the expression of M1AchR, M3AchR, and ADRβ1 under TET2 siRNA and ascorbic acid treatment and found that TET2 deficiency persistently decreased M1AchR, M3AchR, and ADRβ1 expression, similar to the observations of TET2 siRNA alone (Figure 7B–7D). The Western blot analysis also confirmed the alteration of M1AchR, M3AchR, and ADRβ1 expression in *TET2* siRNA-transfected PSGC kl +/+ cells (Figure 7E). Interestingly, TET2 knockdown led to a significant decrease in salivary gland functional markers in PSGC kl +/+ cells. TET2 protein was also inhibited in TET2 siRNA-induced knockdown cells compared with the control cells (Figure 7F). These results suggest that TET2 regulates M1AchR, M3AchR, and ADRβ expression through ascorbic acid in the salivary gland.

We hypothesized that TET2 induces Ach receptor expression by demethylating the CpG island in the promoter region of muscarinic receptor genes. To determine the association between M1AchR gene expression and TET2-induced demethylation, we examined the methylation status of M1AchR promoters by bisulfite sequencing in PSGC kl -/- cells treated with and without ascorbic acid. We identified CpG islands in the range of -2154 to -1850 in the M1AchR promoter region. As shown in Supplementary Figure 2, the total methylation level of the region from -3103 to -984 of M1AchR promoter was 32% in PSGC kl -/- cells without ascorbic acid, while it was changed to 32.7% in ascorbic acid-treated cells. The CpG sites in the promoter region also were not significantly changed, from 0.8% in control cells to 1.0% in ascorbic acid-treated cells. These results suggest that TET2 is indirectly involved in M1AchR expression through DNA demethylation of other specific genes and may be correlated with the expression of salivary gland functional marker genes, such as α-amylase, aquaporin-4, aquaporin-5, and ZO-1.

## DISCUSSION

Although the causes of salivary gland dysfunction in aging are known, we have no adequate therapeutic approaches for salivary gland dysfunction. The studies presented here demonstrate the beneficial therapeutic effect of ascorbic acid on aged salivary gland dysfunction. Prior studies have shown that ascorbic acid deficiency influences salivary flow rates and secretion [22, 23, 27]. However, the direct role of ascorbic acid on salivary gland dysfunction has not been studied. In our previous study, metabolomic profiling of salivary glands in aging-accelerated mice suggested that most metabolic changes in aged salivary glands are attributed to aging. Indeed, we found that aging reduces salivary gland function (loss of salivary secretion) and results in multiple impaired metabolic pathways, including GSH/GSSG and ascorbic acid metabolism, *in vivo* [24]. Understanding the mechanism of metabolic alternation in aging can improve life expectancy and quality of life and promote healthy aging. In this study, we investigated the roles and mechanisms of ascorbic acid on aging-induced salivary gland dysfunction in the extensive aging mouse model of SAMP1/Klotho-/- mice.

First, we found that ascorbic acid and its precursors (glucose 6-phosphate, D-glucuronic acid, and UDP-glucuronic acid) were significantly reduced in SAMP1/Klotho-/- mice compared with wild-type mice. Therefore, these results suggested that the ascorbic acid biosynthesis pathway in the aging SAMP1 kl -/- model may be impaired. It has been suggested that several mammals lack ascorbic acid biosynthesis capacity due to loss-of-function mutations in biosynthetic enzymes such as L-gulono-1,4-lactone oxidase [29]. Additionally, mechanisms for ascorbic acid transport and recycling are important for ensuring the distribution of ascorbic acid in tissues. However, there is no easy explanation for the loss of ascorbic acid biosynthesis capacity during aging. Ascorbic acid is highly concentrated in the brain, such as in the hypothalamus and pituitary and adrenal glands [30]. The acini of salivary glands also have high ascorbic acid levels [27]. This suggests that high levels of ascorbic acid in the brain and salivary gland may correlate with a high redox imbalance and oxidative stress (oxidants and free radicals) for protection against pathological conditions [31]. These biological functions of ascorbic acid are clear, but whether it can be considered to have a role in molecular signaling in the salivary gland function is unknown.

In aging SAMP1 kl -/- mice, supplementation with ascorbic acid significantly increased saliva secretion compared with the control. Ascorbic acid also importantly induced the expression of acetylcholine and/or beta-adrenergic receptors (M1AchR, M3AchR, and Adrb1) and salivary gland functional markers (α-amylase, ZO-1, and Aqua5) *in vitro* and *in vivo*. These results confirmed that ascorbic acid can improve aging-induced salivary gland dysfunction.

The release of acetylcholine from parasympathetic nerves plays a key role in the saliva stimulation–secretion system in salivary acinar cells by acting on muscarinic receptors (mAChRs). Its action is mediated by acinar cell fluid secretion and ductal cell modification of saliva. Acinar cells synthesized and exocytosed the total proteins of saliva [32]. As a previous study showed that ascorbic acid accumulates in acini of salivary glands, we suggest that ascorbic acid may be involved in regulating mAChR expression *in vitro* and *in vivo*. The increased expression of mAChRs by ascorbic acid promotes the release of Ca^2+^ from intracellular organs, ultimately leading to increased salivary gland functional protein and saliva volume. In addition, β-adrenergic receptors activate adenylate cyclase and promote cAMP levels, resulting mainly in protein secretion with relatively little increase in saliva volume [33]. A reduction in ascorbic acid was found in aged salivary glands, which may affect salivary gland function by three possible mechanisms: (1) disturbed release of Ach by Ach regulatory factor malfunction, (2) Ach blockade by central autonomic network (CAN) damage, and (3) impaired glucose synthesis and energy homeostasis. Glucose is an essential energy source for adult human tissues, including the brain. Ascorbic acid is synthesized directly from the glucose metabolic pathway in most animals. Ascorbic acid also participates as a metabolic switch modulating glucose and lactate metabolism in neurons. The mechanism of acetylcholine, a neurotransmitter of the parasympathetic nervous system, in salivary gland function was demonstrated by the findings of our previous studies in SAMP1/Klotho -/- mice as an animal model of aging.

Recent findings support that ascorbic acid can regulate the methylation status of DNA to improve oocyte maturation and development [34]. The epigenetic modification of DNA, such as DNA methylation/demethylation, alters DNA secondary structure and regulates patterns of gene expression. DNA methylation is removed through demethylation. An active mechanism of DNA demethylation by the ten-eleven translocation methylcytosine dioxygenase (TET) has been reported [35]. Studies have confirmed that ascorbic acid regulates TET to dynamically modulate the epigenetic status of DNA/histone methylation. Mechanistically, in the proposed model, ascorbic acid converts Fe^3+^ into Fe^2+^, thus providing the necessary Fe^2+^ ion to activate the TET family proteins [28]. Therefore, we hypothesized that ascorbic acid could modulate mAChRs and/or salivary gland function through epigenetic modulation by TET proteins.

In this study, among the TET family, only TET2 was detected in salivary glands, while TET1 and TET3 were not detected (data not shown). Compared with SAMP1/kl +/+ mice, SAMP1/kl -/- mice had significantly reduced TET2 mRNA expression by more than 80% in the salivary gland. Treatment with ascorbic acid significantly induced TET2 expression and DNA demethylation in SAMP1/kl-/- mice. In addition, TET2 overexpression significantly upregulated M1/M3AchR and salivary gland functional protein expression in PSGC kl +/+ cells. Generally, activation of the demethylation process can rescue injury, promote regeneration, maintain normal gene expression, and reverse aging-induced damage. For example, retina nerve supply and vision loss in aged mice can be recovered with Oct4-Sox2-Klf4-induced epigenetic reprogramming, which depends on TET1 and TET2 [36]. In the salivary gland, the demethylation agent decitabine can induce saliva secretion in aged mice through the water channel Aqua5 [37]. Additionally, overexpression of TET2 can rescue functional cognition and induce neurogenesis in the mouse brain [38]. Taken together, we demonstrated that ascorbic acid induces global demethylation and salivary gland functional gene expression by enhancing TET2 expression/activity in salivary glands. We also concluded that TET2 exhibits a high potential in recovering aging-induced damage in salivary gland dysfunction.

Using bisulfite sequencing analysis, we examined the methylation status of the M1AchR prompter CpG island in PSGCs after treatment with or without ascorbic acid. The 5-methylcytosine content showed no significant difference in methylation status between ascorbic acid-treated cells and untreated cells. In PSGC kl -/- cells, the total methylation level of the M1AchR promoter was 32% without ascorbic acid, while it was 32.7% in ascorbic acid-treated cells. Therefore, M1AchR expression is not correlated with the methylation status of the M1AchR promoter region. In this study, we showed that the methylation status of the M1AchR prompter CpG island does not occur directly through the ascorbic acid-induced TET2 protein. However, it is necessary to investigate DNA methylation at other acetylcholine or beta-adrenergic receptor (M3AchR and Adrb1) promoters.

This suggests that alternative mechanisms might be responsible for the induction of M1AchR protein expression by ascorbic acid *in vitro* and *in vivo*. Among many genes altered in DNA methylation by ascorbic acid-induced TET2 expression/activation, the demethylation of M1AchR regulator proteins may be involved in acetylcholine or beta-adrenergic receptor expression. Further studies are required to fully explain the mechanism of TET2-induced recovery. Additionally, whether decreased levels of ascorbic acid are responsible for salivary gland dysfunction in aging is an unsolved issue, but the effects of ascorbic acid supplementation on salivary gland functional mechanism provide more insight about potential therapy to revert aging-induced dysfunction.

**Figure 8:**
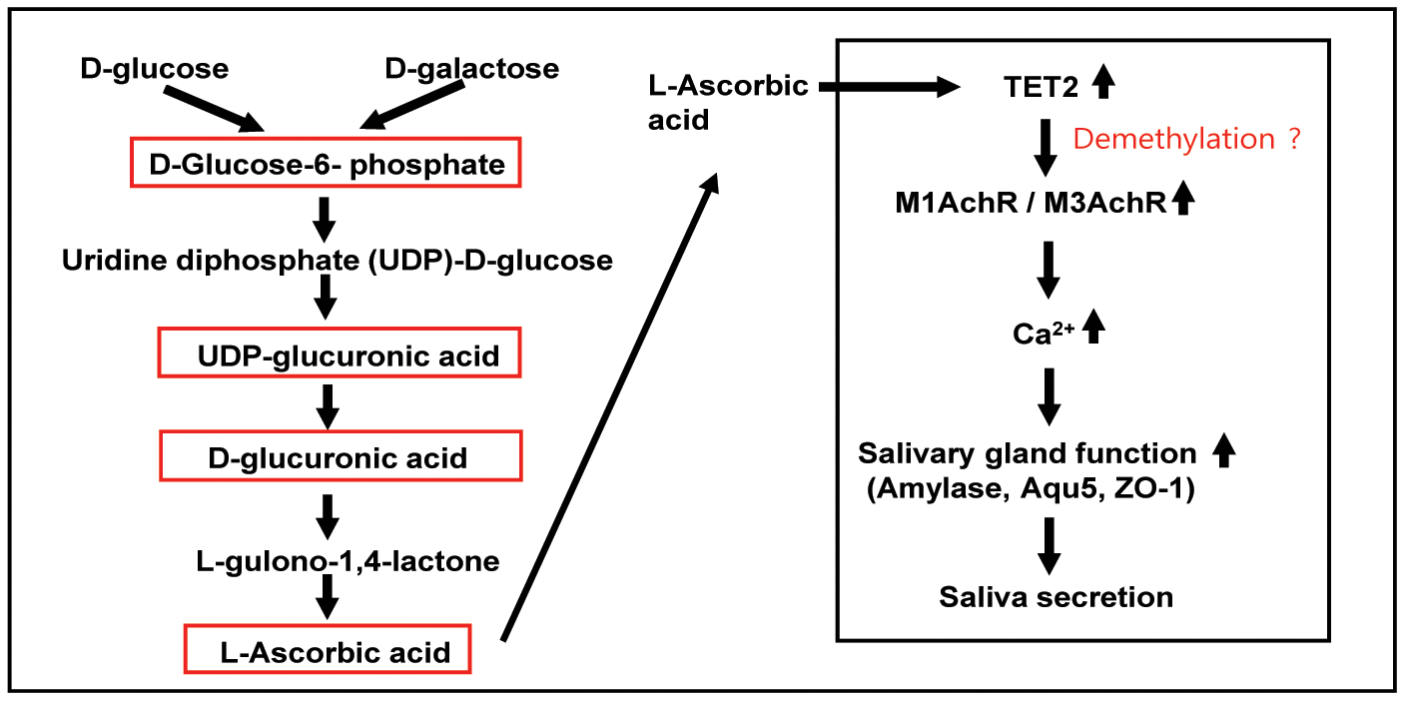
**Schematic pathway for the metabolism and roles of ascorbic acid in salivary glands.** In most mammals, glycogenolysis, as a major source of UDP-glucuronic acid, leads to ascorbic acid synthesis. The glycogenolysis pathway is involved in the metabolism of glucose under normal and disease conditions and in the regulation of physiological functions. D-Glucose is converted into L-ascorbic acid via D-glucuronic acid, L-gulonic acid, and 2-keto-L-gulonolactone as intermediates. Ascorbic acid stimulates TET2 activity by promoting the recycling of inactive oxidized ferric iron (Fe^3+^) to actively reduced ferrous iron (Fe^2+^) *in vitro* and *in vivo*. Activation/expression of TET2 is indirectly involved in the expression of muscarinic and adrenergic receptors in salivary glands through global DNA demethylation. Ascorbic acid-induced TET2 promotes the expression of muscarinic and adrenergic receptors, which induce Ca^2+^ signaling, thereby inducing salivary gland functional protein and ultimately maintaining saliva secretion.

In conclusion, our study demonstrated that the biosynthesis pathway of ascorbic acid in the accelerated aging mouse model SAMP1/kl-/- mice is impaired, with the depletion of the intermediate metabolites G6P, UDP-glucuronic acid, and D-glucuronic acid and the final product L-ascorbic acid. Supplementation with ascorbic acid significantly increased saliva volume and salivary gland functional proteins (α-amylase, ZO-1, and Aqua5), probably through upregulation of TET2 and induction of a demethylation process to upregulate salivary gland receptors (Figure 8). Our results will contribute to a better understanding of aging-related ascorbic acid metabolic alterations and provide more insight into potential therapies to revert aging-induced dysfunction.

## MATERIALS AND METHODS

### Cell cultures and reagents

Wild-type primary salivary gland cells (PSGC kl +/+) and mutant primary salivary gland cells (PSGC kl -/-) were generated as previously described [39]. The PSGC kl +/+ and PSGC kl -/- cell lines were cultured in Dulbecco’s modified Eagle’s medium (DMEM) (Welgene Inc., Gyeongsanbuk-do, Korea) containing 10% fetal bovine serum (FBS), 100 units/mL penicillin, and 100 μg/mL streptomycin. Cells were maintained in a 5% CO_2_ humidified atmosphere at 37° C.

### DNA construction and siRNA transfection assay

pcDNA3-TET2 was a gift from Yi Zhang (Addgene plasmid # 60939) [40]. Plasmid transfection was performed using FuGENE® 6 Transfection Reagent (Promega, Madison, WI, USA) according to the manufacturer’s protocol. siRNAs constructed for mouse ChAT were obtained from Bioneer (Deajeon, Korea). siRNA transfection was performed using Lipofectamine 2000 (Thermo Fisher Scientific, Waltham, MA, USA) following the manufacturer’s protocol. Briefly, cells were seeded at 10^5^ cells/well in a 6-well plate 24 hrs before plasmid/siRNA transfection. Cells were harvested 48 hrs post-transfection, and subsequent experiments were conducted.

### Quantitative reverse transcription polymerase chain reaction (qRT–PCR) analysis

Total RNA was extracted from the transfected cells using TRIzol reagent (Takara Bio Inc., Shiga, Japan) according to the manufacturer’s instructions. RNA quality was determined by OD280/260 to ensure that the RNA template had a 260/280 ratio between 1.8-2.0. Reverse transcription was performed on 1 μg of total RNA using oligo dT primers and M-MLV Reverse Transcriptase (Invitrogen) in a final volume of 20 μL for 5 min at 65° C followed by 1 hr at 37° C. Quantitative reverse transcription PCR was performed using the GoTaq® 1-Step RT–qPCR System kit (Promega, Madison, WI, USA) according to the manufacturer’s protocol. The primer sets used are shown in Table 1. The relative expression level of target genes is represented by the 2-ΔΔCt value [41].

**Table 1:** Primer sequences for qRT-PCR.

**Gene**	**Sequence**
M1AchR	Forward: 5’– TCTCTGAATGCTGGAAGTAAAGA – 3’
	Reverse: 5’– GAGACCCTAGATTCAGTCCCA – 3’
M3AchR	Forward: 5’– AGGGCTGACTACTTAATCTTGGATA – 3’
	Reverse: 5’– TGCAAGGTCATTGTGACTCTC – 3’
Adrb1	Forward: 5’– GAGCAGTTCGAAGACCTGCTTGTG – 3’
	Reverse: 5’– CCTGTCAGGATGGACTGGTCGA – 3’
Adrb2	Forward: 5’– GGGAACGACAGCGACTTCTT – 3’
	Reverse: 5’– AAGTCCAGAACTCGCACCAG – 3’
TET2	Forward: 5’– AGAGCCTCAAGCAACCAAAA – 3’
	Reverse: 5’– ACATCCCTGAGAGCTCTTGC – 3’
α-Amylase	Forward: 5’– GGTGCAACAATGTTGGTGTC – 3’
	Reverse: 5’– ACTGCTTTGTCCAGCTTGAG – 3’
ZO-1	Forward: 5’– CGAGGCATCATCCCAAATAAGAAC – 3’
	Reverse: 5’– TCCAGAAGTCTGCCCGATCAC – 3’
Aqua5	Forward: 5’– CGACCGTGTGGCTGTGGTCA – 3’
	Reverse: 5’– GTGCCGGTCAGTGTGCCGTC – 3’
GAPDH	Forward: 5’– AGCCAAAAGGGTCATCATCTCTGC – 3’
	Reverse: 5’– CCTTCCACAATGCCAAAGTTGTCA – 3’

Primers for qRT-PCR were designed by Primer-Blast tools of NCBI.

### Western blot analysis

Cells were lysed using radioimmunoprecipitation assay (RIPA) buffer (Biosesang, Seongnam, Korea) containing a protease inhibitor cocktail (1 μg/mL) and phosphatase inhibitor (1 μg/mL). Then, 15 μg of cell lysates were separated by 10% sodium dodecyl sulfate-polyacrylamide gel electrophoresis (SDS–PAGE) and transferred to a polyvinylidene difluoride (PVDF) membrane (Millipore, Burlington, NJ, USA). The membranes were blocked with 5% skim milk for 2 h and then incubated overnight at 4° C with primary antibodies (1:1000, diluted in TBST). Primary antibodies against actin (sc-47778), amylase (sc-514313), M1AchR (sc-365966), and M3AchR (sc-518107) were purchased from Santa Cruz Biotechnology (Santa Cruz, CA, USA). The tight junction proteins zonula occludens-1 (ZO-1) and ten-eleven translocation 2 (TET2) were detected with anti-ZO-1 antibody (21773-1-AP) and anti-TET2 antibody (21207-1-AP), respectively, from Proteintech (Rosemont, IL, USA). The water channel protein Aqp5 was detected by an anti-aquaporin 5 antibody (AB15858) from Merck Millipore (Burlington, NJ, USA). The primary antibody against adrenergic receptor β1 (bs-20177R) was purchased from Bioss (Woburn, MA, USA). All primary antibodies were diluted in TBS+0.1% Tween 20 (TBST) at a 1:1000 ratio. On the next day, the membranes were washed 3 x 5 minutes with TBST and then incubated with appropriate secondary antibodies conjugated to HRP (Promega, Madison, WI, USA) for 1 hour at room temperature. The protein signal was visualized by Immobilon® Western Chemiluminescent HRP Substrate reagents (Merck Millipore, Burlington, NJ, USA) through a luminescence image analyzer (LAS-1000, Fujifilm, Tokyo, Japan).

### Immunohistochemistry and immunocytochemistry assay

Salivary glands harvested from animals were fixed overnight in 10% neutral buffered formalin and embedded in paraffin. Tissue sections (4 μm thick) were stained using the avidin-biotin-peroxidase complex (ABC) immunohistochemical method, following the protocol of the Vectastain® ABC HPR kit PK-4000 from Vector Labs (Burlingame, CA, USA). Briefly, tissue sections were deparaffinized in a series of ethanol before being treated with 0.3% H_2_O_2_ in methanol for 10 min to block endogenous peroxidase activity. Then, antigen retrieval was conducted by heating the tissue sample at 90° C for 20 min in 10 mM citrate buffer (pH 6.0), followed by gradual cooling for 20 min. The tissue was blocked in PBS + 2% normal goat serum for 1 h and washed with PBS 3x15 mins. Tissue samples were then incubated with primary antibody solution (1:200, diluted in blocking solution) at 4° C overnight. The same antibody used in the Western blot analysis was used to detect the target proteins. On the next day, tissue samples were incubated with anti-mouse biotinylated secondary antibody (1:200, diluted in blocking solution) for 1 hour at RT, followed by incubation with an avidin-biotin complex solution and 3,3-diaminobenzidine (DAB) solution (SK-4100, Vector Labs, Burlingame, CA, USA) before mounting. Images were taken under bright light using an Olympus BX50 inverted microscope (Olympus Corporation, Tokyo, Japan). For the immunocytochemistry assays, samples were fixed with 10% neutral buffered formalin for 10 min at RT and then permeabilized with PBS+0.2% Triton X-100 (PBSTx) for 30 min at RT. Fixed samples were incubated with primary antibodies (diluted in PBSTx+5% normal goat serum, 1:200) overnight at 4° C, washed 3 x 5 min in PBSTx at RT, incubated with secondary antibodies (diluted in PBSTx+5% normal goat serum, 1:200), washed 3 x 5 min in PBST at RT, and counterstained with 4′,6-diamidino-2-phenylindole (DAPI, 300 nM, 5 minutes) before mounting. The same primary antibodies as listed above in the Western blot analysis section were used. Samples were visualized with a confocal microscope (Nikon Corporation, Tokyo, Japan).

### DNA methylation assay (5-methylcytosine quantification)

The global DNA methylation levels were determined by measuring the 5-methyl-cytosine level with a Global DNA Methylation competitive enzyme-linked immunosorbent assay (ELISA) kit from Cell Biolabs (San Diego, CA, USA). Briefly, genomic DNA was extracted from cell samples using the Wizard® Genomic DNA purification kit (Promega, Madison, WI, USA). A total of 1 μg genomic DNA (0.1 μg/μL concentration) was denatured into single-stranded DNA by heating the samples at 95° C for 10 mins, followed by quick cooling on ice. Next, 1 μL of nuclease P1 (New England Biolabs, Ipswich, MA, USA) and an appropriate amount of nuclease P1 reaction buffer (New England Biolabs, Ipswich, MA, USA) were added to single-stranded DNA samples, and the whole mixture was incubated at 95° C for 1 hr to digest DNA into nucleosides. After that, the samples were treated with calf intestinal alkaline phosphatase (Takara Bio Inc., Shiga, Japan) for 1 hr at 37° C. Further steps were conducted according to the manufacturer’s recommendation. Sample absorbance was measured at 450 nm using a microplate reader (DTX880, Beckman Coulter, Inc., California, USA).

### Bisulfite conversion and bisulfite sequencing

Briefly, genomic DNA was extracted from cell samples using the Wizard® Genomic DNA purification kit (Promega, Madison, WI, USA). One microgram of genomic DNA was subjected to bisulfite conversion using Protocol A of the EpiJET Bisulfite Conversion Kit (K1461, Thermo Fisher Scientific). Bisulfite-converted DNA was subjected to bisulfite sequencing PCR using the primers listed in Supplementary Table 1. Nested PCR was used to increase the specificity. Primers were designed using the MethPrimer prediction software [42]. PCR products were purified from gels by a QIAquick gel extraction kit (Qiagen) and cloned into the pGEM-T vector using the TA cloning method. DNA plasmids from 5 individual colonies were extracted using the Wizard® Plus SV Minipreps DNA Purification kit (Promega, Madison, WI, USA) and subjected to sequencing using Sanger sequencing services (Macrogen, Inc., Korea). Sequencing results were analyzed using QUMA (quantification tool for methylation analysis) tools at http://quma.cdb.riken.jp/ [43].

### Animal handling

Aging SAMP1/kl-/- mice were generated as previously described [26]. A total of 9 SAMP1/kl -/- mice (4 weeks old, weight 7–10 g) were used in the present study. The animals were housed in a controlled environment with a temperature of 25 ± 1° C and a humidity of 50 ± 5%. Food and water were provided ad libitum. All animal procedures were performed under a protocol approved by the Chosun University Institutional Animal Care and Use Committee (CIACUC2020-S0025). Ascorbic acid (100 mg/kg/day) was dissolved in distilled water and orally administered by gavage for 18 days. Control samples were provided with saline.

### Statistical analysis

All experiments were performed at least in triplicate. The results are expressed as the mean ± standard deviation (SD). To determine the significant difference between the control and experimental groups, Student’s t-test was applied. *P* values of less than 0.05 were considered statistically significant.

## Supplementary Material

Supplementary Figures

Supplementary Table 1
