# Supplementary material for: Ascorbic acid induces salivary gland function through TET2/acetylcholine receptor signaling in aging SAMP1/Klotho (-/-) mice
Source: Aging (Albany NY). 2022 Aug 11;14(15):6028–46. doi: 10.18632/aging.204213 (PMC9417236; doi:10.18632/aging.204213)
Supplement: Supplementary Figures [file aging-14-204213-s001.pdf]

# SUPPLEMENTARY FIGURES

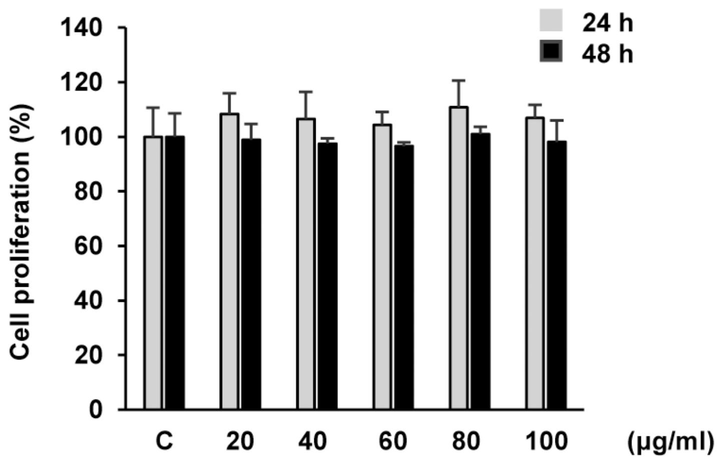


**Supplementary Figure 1. Effect of ascorbic acid on PSGC kl+/+ cell proliferation.** PSGC kl +/+ cells were treated with ascorbic acid (20-100 μg/ml) for 24 or 48 hours. Cell proliferation was evaluated by MTT assay.

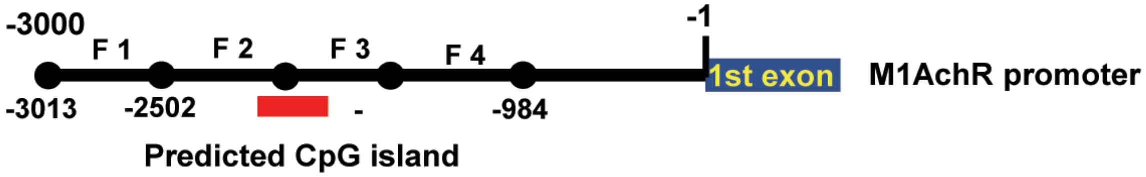


**Total Methylation rate (%)**

| | Fragment 1 | Fragment 2-3 (CpG island ) | Fragment 4 | Total |
|---|---|---|---|---|
| Untreated | 77.1 | 0.8 | 18.2 | 32 |
| AA treated | 78.8 | 1.0 | 18.2 | 32.7 |

**Supplementary Figure 2. Evaluation of the DNA methylation level in the M1AchR promoter CpG region.** PSGC kl -/- cells were treated with ascorbic acid for 24 hr. After genomic DNA isolation, the methylation status of the promoter of M1AchR was analyzed by using bisulfite sequencing. Numbers with a minus sign indicate the position of the fragment (F1-F4) relative to the transcription starting site. The red box represents a CpG island in the M1AchR promoter. The percentage of methylation indicates the proportion of methylated fragments, including CpG regions.
