# Supplementary material for: Ascorbic acid induces salivary gland function through TET2/acetylcholine receptor signaling in aging SAMP1/Klotho (-/-) mice
Source: Aging (Albany NY). 2022 Aug 11;14(15):6028–46. doi: 10.18632/aging.204213 (PMC9417236; doi:10.18632/aging.204213)
Supplement: Supplementary Table 1 [file aging-14-204213-s002.pdf]

SUPPLEMENTARY TABLE

Supplementary Table 1. Primer sequences for bisulfite sequencing.

Primer name	Sequence (5' -> 3')
Inner Primer (F) - Cpg Island	TTTATTTGAATAGAGGGGAAAAATG
Inner Primer (R) - CpG island	TAAATCTCCTCATACCAATTAAAAA
Outer Primer (F) - CpG islands	TGATTAAAAATAGTAATTAGTAGGAG
Outer Primer (R) - CpG islands	TTCTAAAATCTATACAACTACAAAAA
Inner Primer (F) - Fragment 1	ATGTTTTATTTTATGGAGGTTAAGG
Inner Primer (R) - Fragment 1	AATACTAAATACTTACTTCCACCAA
Outer Primer (F) - Fragment 1	GAAGGTTTAGTGGGGTTTTAAATTT
Outer Primer (R) - Fragment 1	CATCAACCAACACTTTCAACATCTA
Inner Primer (F) - Fragment 4	ATGGTTTTTTTAATATGTAGTTTGT
Inner Primer (R) - Fragment 4	CTCCTATATATTTTACCCCATAAAC
Outer Primer (F) - Fragment 4	AAAGAGGAAGTTTGATTTAGATTTG
Outer Primer (R) - Fragment 4	ATAAAAACCTAAATCCAACAATACC

Nested PCR was used to amplify the bisulfite-converted DNA to improve the efficiency and specificity of the cloning process. Each fragment was amplified with the “Outer” primer set (1st round); then 5 µl of the PCR products was used for the 2nd round of amplification with the “Inner” primer set. After 2 rounds of amplification, the PCR products were loaded on agarose gel 1% and then purified and cloned into pGEM-T vectors, before sequencing and methylation analysis, as mentioned above in the material and method section.
